# Association of circulating omentin-1 level with arterial stiffness and carotid plaque in type 2 diabetes

**DOI:** 10.1186/1475-2840-10-103

**Published:** 2011-11-22

**Authors:** Hye Jin Yoo, Soon Young Hwang, Ho Cheol Hong, Hae Yoon Choi, Sae Jeong Yang, Ji A Seo, Sin Gon Kim, Nan Hee Kim, Kyung Mook Choi, Dong Seop Choi, Sei Hyun Baik

**Affiliations:** 1 Division of Endocrinology and Metabolism, Department of Internal Medicine, Korea University College of Medicine, Seoul, Korea; 2 Department of Biostatistics, Korea University College of Medicine, Seoul, Korea

**Keywords:** omentin-1, pulse wave velocity, carotid intima media thickness, type 2 diabetes

## Abstract

**Background:**

Adipokines contribute directly to the atherosclerotic process, connecting metabolic disorders such as obesity and diabetes to cardiovascular disease. Omentin-1 is a recently discovered novel adipokine, so data about the relationship of this adipokine to vascular health in type 2 diabetes is limited.

**Methods:**

We enrolled 60 people with type 2 diabetes, with or without carotid plaque, and 30 participants with normal glucose tolerance. We measured serum omentin-1, high-sensitivity C-reactive protein (hsCRP) levels, and the homeostasis model assessment of insulin resistance (HOMA-IR), as well as other cardiovascular risk factors. Vascular health was assessed by brachial ankle pulse wave velocity (baPWV) and carotid intima-media thickness (IMT).

**Results:**

Serum omentin-1 levels were significantly decreased in type 2 diabetes patients compared to normal glucose controls and was further reduced in type 2 diabetes patients with carotid plaque compared to those without carotid plaque. Multiple stepwise regression analysis showed that age, systolic blood pressure, history of use of statins, angiotensin receptor blockers or angiotensin-converting enzyme inhibitors, and serum omentin-1 level were independent factors determining baPWV in people with type 2 diabetes (*r*^2 ^= 0.637). Furthermore, in multivariate logistic regression analysis, circulating omentin-1 level was an independent decisive factor for the presence of carotid plaque in type 2 diabetes patients, even after adjusting for age, gender, body mass index, systolic blood pressure, fasting blood glucose, low density lipoprotein cholesterol, and history of smoking and medication (odds ratio, 0.621; 95% confidence interval, 0.420-0.919; *P *= 0.017).

**Conclusions:**

Circulating omentin-1 level was independently correlated with arterial stiffness and carotid plaque in type 2 diabetes, even after adjusting for other cardiovascular risk factors and detailed medication history.

## Background

The prevalence of diabetes is expected to rapidly increase from 171 million individuals (2.8% of the world's population) in 2000 to 366 million (4.4% of the world's population) by 2030 [1]. Individuals with diabetes have two- to three-fold increased risk of myocardial infarction or stroke compared to individuals without diabetes [2]. However, the underlying mechanisms linking type 2 diabetes with cardiovascular disease remain poorly understood. Recent evidence has shown that some adipokines are major regulators of insulin resistance and direct mediators of endothelial dysfunction and macrophage infiltration of vessel walls [3]. The identification of a novel adipokine involved in the atherosclerotic process might provide new opportunities for preventing cardiovascular disease in patients with type 2 diabetes.

Omentin is a novel fat depot-specific adipokine that was identified in 2003 from a visceral omental adipose tissue cDNA library [4]. The omentin gene is located in the 1q22-q23 chromosomal region, which has been linked to type 2 diabetes in several populations [5,6], suggesting that omentin may be a candidate gene for type 2 diabetes susceptibility in humans. In fact, Yang et al. [7] demonstrated that recombinant omentin-1 enhances insulin-stimulated glucose uptake and Akt phosphorylation in human adipocytes. Several clinical studies have shown that the serum omentin-1 level is significantly decreased in obese patients or patients with polycystic ovary syndrome or diabetes [8-10]. In addition, circulating omentin-1 levels are negatively correlated with metabolic risk factors, including body mass index, waist circumference, and insulin resistance [8-10].

Recent clinical studies have focused on the influence of the novel adipokine omentin-1 on vascular health [11-14]. In these studies, circulating omentin-1 had a negative correlation with atherosclerotic parameters. However, no previous studies have clarified the relationship of serum omentin-1 with atherosclerosis in subjects with type 2 diabetes. Carotid intima-media thickness (IMT) and arterial stiffness are useful surrogate markers for subclinical atherosclerosis and are significantly correlated with various metabolic risk factors [15,16]. In prospective studies, both arterial stiffness and carotid IMT have proven to be consistent and independent predictors for cardiovascular events [17]. Recently, Dall Pozza et al. [18] showed that longitudinal carotid IMT is indicative of disease progression during a four year period, even in adolescents with type 1 diabetes, and that brachial ankle pulse wave velocity (baPWV) is a non-invasive method for quickly measuring arterial stiffness that is not influenced by operator technique [19].

Therefore, in this study, we determined the relationship of circulating omentin-1 levels with atherosclerosis, as measured by carotid IMT and baPWV, in subjects with type 2 diabetes and controls. Furthermore, to determine the exact correlation of circulating level of omentin-1 with baPWV and carotid IMT in type 2 diabetes, we adjusted for other cardiovascular risk factors and detailed drug history that could affect vascular function.

## Methods

### Study design and participants

The study subjects were 30 people with type 2 diabetes without carotid plaque, 30 people with type 2 diabetes with carotid plaque, and 30 control participants with normal glucose tolerance. Diabetes was defined as a fasting plasma glucose value ≥ 7.0 mmol/L (126 mg/dL) or a previous diagnosis of diabetes. All were self-referred from the Department of Endocrinology and Metabolism at Korea University Guro Hospital. All subjects in the control group were administered a 75-g oral glucose loading test to exclude subjects with pre-diabetes or undiagnosed diabetes using the criteria of the American Diabetes Association [20]. The study exclusion criteria included histories of cardiovascular diseases (myocardial infarction, unstable angina, stroke, peripheral artery disease, or cardiovascular revascularization), stage 2 hypertension (resting blood pressure ≥ 160/100 mmHg), uncontrolled diabetes mellitus (HaA1c > 10%), malignancy, or severe renal or hepatic disease. We also excluded patients with inflammatory conditions such as vasculitis or aortitis, or acute infectious disease. We investigated smoking and medication histories including use of insulin, oral hypoglycemic agents, statins, anti-platelet agents, angiotensin receptor blocker (ARB), angiotensin-converting enzyme (ACE) inhibitors or calcium channel blockers. All participants provided written informed consent, and the Korea University Institutional Review Board, in accordance with the Declaration of Helsinki of the World Medical Association, approved the study protocol.

### Clinical and laboratory measurements

Body mass index (BMI) was calculated as weight/height^2 ^(kg/m^2^) and waist circumference was measured at the midpoint between the lower border of the rib cage and the iliac crest. All blood samples were obtained the morning after a 12-hour overnight fast, and were immediately stored at -80°C. Serum triglycerides and high-density lipoprotein-cholesterol (HDL-C) were determined enzymatically using a chemical analyzer (Hitachi 747; Hitachi Inc., Tokyo, Japan). Low-density lipoprotein (LDL)-cholesterol concentration was estimated using the Friedewald formula [21]. The glucose oxidase method was used to measure plasma glucose and an electrochemiluminescence immunoassay (Roche Diagnostics, Indianapolis, IN, USA) was used to measure insulin levels. Insulin resistance was calculated by the homeostasis model assessment of insulin resistance (HOMA-IR). hsCRP levels were measured by latex-enhanced turbidimetric immunoassay (HiSens hsCRP LTIA, HBI Co., Ltd.), with an interassay coefficient of variation of 7.2%. Serum concentrations of omentin-1 were determined by an enzyme-linked immunosorbent assay (ELISA) kit (Apotech, Axxora, Nottingham, UK); intra- and inter-assay variations were 3.6 ± 0.7% and 4.6 ± 0.3%, respectively.

### Measurement of baPWV

After subjects rested in the supine position for 5 minutes, baPWV was measured using a volume-plethysmographic apparatus (model BP-203RPE II; Colin, Komaki, Japan) that simultaneously recorded baPWV and brachial and ankle blood pressures on the left and right sides. The details of this method, including validity and reproducibility, were described previously by Yamashina et al. [22]. The intra- and inter-observer reproducibility of this method in the present study were 10.0% and 8.4%, respectively. The baPWV was calculated as the mean of the left and right baPWV values.

### Measurement of carotid IMT

The IMT of the common carotid artery was determined using high-resolution B-mode ultrasonography (EnVisor; Philips Medical Systems, Andover, MA, USA) with a 5-12 MHz transducer. Carotid plaque was defined as a focal structure protruding into the arterial lumen with a thickness ≥ 1.3 mm [23]. Measurements of the carotid IMT were made using IMT measurement software (Intimascope; Media Cross Co., Tokyo, Japan) at three levels of the lateral and medial walls of the carotid artery, 1-3 cm proximal to the carotid bifurcation. The maximal IMT was the value at the maximal point of the region. Carotid IMT was calculated as the mean of the maximal left and right IMT values. All measurements were recorded by a single trained technician who was blinded to the anthropometric and laboratory data.

### Statistical analysis

Each variable was examined for normal distribution by the Kolmogorov-Smirnov equality of distributions test. Data are expressed as the mean with standard deviations (SDs) or median (interquartile range, 25%-75%), or as a percentage. Differences among the groups were tested using ANOVA for normally distributed variables or the Kruskal-Wallis H test for non-normally distributed variables, and Fisher's exact test or Pearson's chi-square test for categorical variables. Subsequent pair-wise comparisons were performed by Tukey's HSD post hoc analysis or the Wilcoxon rank-sum test. Analysis of covariance (ANCOVA) was used to compare the mean baPWV values according to tertile of serum omentin-1 level after adjusting for known covariates. Multiple linear stepwise regression analysis with baPWV as a dependent variable was performed to identify the risk factors that determined baPWV in type 2 diabetes. Subsequently, age, gender, BMI, systolic blood pressure, fasting blood glucose, smoking and medication history, including usage of statins and ARB or ACE-inhibitors, and circulating omentin-1 level, were used as independents variables. Unadjusted and adjusted odds ratios (ORs) with 95% confidence intervals (CIs) predicting carotid plaque in type 2 diabetes patients based on circulating omentin-1 level were generated using univariate and multivariate logistic regression analyses after controlling for other potential covariates. All statistical results were based on 2-sided tests. Data were analyzed using SPSS for Windows (version 12.0; SPSS Inc., Chicago, IL, USA). We regarded a *P *value of less than 0.05 as statistically meaningful.

## Results

### Characteristics of study subjects

**Figure 1:**
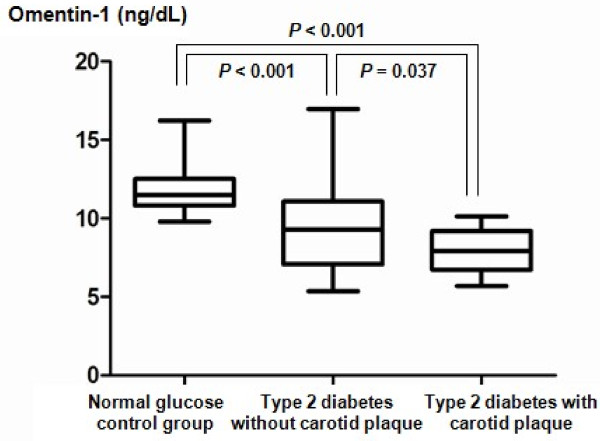
**Serum omentin-1 levels in normal glucose tolerance group, type 2 diabetes patients without carotid plaque and type 2 diabetic patients with carotid plaque**.

The clinical and biochemical characteristics of the study subjects are in Table 1. Although there were no significant differences in age, gender or BMI, significant differences between groups were seen in waist circumference, systolic blood pressure, fasting blood glucose, HOMA-IR, and history of medication use including statins, anti-hypertensive agents, and anti-platelet agents (Table 1). In particular, baPWV values showed an increasing trend in subjects with normal glucose tolerance, those with type 2 diabetes without plaque and those with type 2 diabetes with plaque. In addition, circulating omentin-1 levels were significantly decreased in type 2 diabetes patients compared to normal glucose controls and were further reduced in type 2 diabetes patients with carotid plaque compared to those without carotid plaque (Figure 1).

**Table 1:** Baseline characteristics of study subjects

	Normal glucose tolerance		Type 2 diabetes		Type 2 diabetes		*P*-value
			without carotid plaque		with carotid plaque		
	(N = 30)		(N = 30)		(N = 30)		
Age (years)	54.07	± 8.14	53.1	± 6.81	56.47	± 6.04	0.169
Gender (M:F)	12	: 18	10	: 20	15	: 15	0.418
BMI (kg/m^2^)	23.99	± 2.88	23.89	± 2.29	24.18	± 2.11	0.895
Waist circumference (cm)	85.19	± 6.05^a^	93.68	± 6.76^b^	95.12	± 3.98 ^b^	< 0.001
SBP (mmHg)	119.67	± 12.73 ^a^	119.33	± 10.56 ^a^	129.83	± 17.95 ^b^	0.007
DBP (mmHg)	77.67	± 10.06	74.5	± 8.55	77.72	± 9.92	0.332
Fasting blood glucose (mmol/L)	5.16	± 0.55 ^a^	7.06	± 2.06 ^b^	7.28	± 1.53 ^b^	< 0.001
HOMA_IR	1.66	(0.92, 2.52) ^a^	2.7	(1.91, 4.10) ^b^	2.96	(1.92, 3.86) ^b^	0.002
HbA1c	5.68	± 0.34 ^a^	7.27	± 1.04 ^b^	7.4	± 0.94 ^b ^	< 0.001
LDL cholesterol (mmol/L)	2.58	± 0.81	2.28	± 0.60	2.27	± 0.50	0.122
HDL cholesterol (mmol/L)	1.41	± 0.39	1.24	± 0.34	1.29	± 0.27	0.159
Triglyceride (mmol/L)	1.02	(0.70, 1.56)	1.17	(0.98, 2.29)	1.36	(1.02, 1.68)	0.057
hsCRP (mg/dL)	0.56	(0.34, 1.45)	0.57	(0.35, 1.21)	0.45	(0.30, 1.13)	0.428
Carotid IMT (cm)	0.84	± 0.11 ^a^	0.86	± 0.12 ^a^	0.98	± 0.16 ^b^	< 0.001
baPWV (m/sec)	13.26	± 1.77 ^a^	14.29	± 2.57 ^a^	16.22	± 3.07 ^b^	< 0.001
Current smoker (%)	26.77		30		46.67		0.218
Statin (%)	10.00 ^a^		33.33 ^ab^		40.44 ^b^		0.024
Anti-platelet agent (%)	6.67		6.67		26.67		0.031
ARB or ACE inhibitor (%)	10.00 ^a^		16.67 ^ab^		40.00 ^b^		0.013
Calcium channel blocker (%)	10		13.33		20		0.533
Insulin (%)	-		13.33		16.66		0.718
Sulfonylurea (%)	-		33.33		43.33		0.426
Glucophage (%)	-		53.33		56.66		0.795
Thiazolidinedione (%)	-		10		13.33		0.688
Duration of diabetes (years)	-		8.47	± 5.46	12.17	± 7.35	< 0.001

Data are expressed as the mean with SD, median (inter-quartile range), or percentage. *P*-value for overall difference among the groups was calculated from the analysis of variance (ANOVA), the Kruskal-Wallis H test, or Pearson's chi-square test. BMI, body mass index; SBP, systolic blood pressure; DBP, diastolic blood pressure; HOMA-IR, homeostasis model assessment of insulin resistance; LDL, low-density lipoprotein; HDL, high-density lipoproteins; hsCRP, high sensitivity C-reactive protein; IMT, intima-media thickness; baPWV, brachial ankle pulse wave velocity; ARB, angiotensin receptor blocker; ACE, angiotensin-converting enzyme inhibitor.

^a, b, c^: same letter indicates no statistical difference between the groups based on post hoc analysis.

### Arterial stiffness stratified by the tertile of serum omentin-1 level in type 2 diabetes

**Figure 2:**
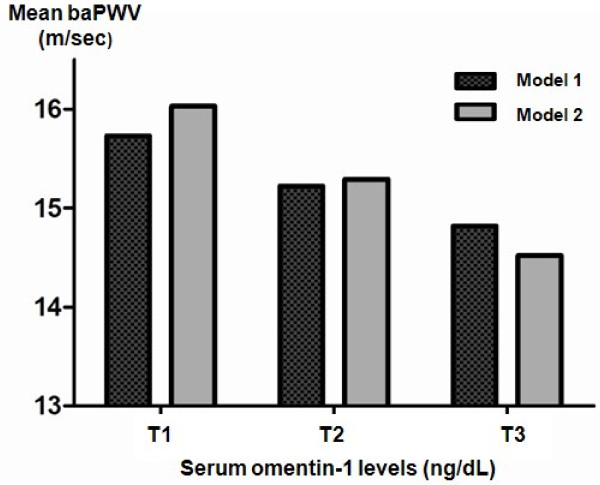
**Mean values of brachial ankle pulse wave velocity (baPWV) based on the tertile of serum omentin-1 level in type 2 diabetes (N = 60)**. T1: lowest tertile of serum omentin-1, T2: median tertile of serum omentin-1, T3: highest tertile of serum omentin-1. Model 1: adjusted for age, gender and body mass index. Model 2: adjusted for age, gender, body mass index, systolic blood pressure, smoking and medication history including statins, angiotensin receptor blockers, or angiotensin-converting enzyme inhibitors.

Subjects with the highest tertile serum levels of omentin-1 (T3) exhibited the most significant detrimental signs of baPWV compared to those in tertile serum omentin-1 groups T2 or T1 after adjusting for age, gender, and BMI (Figure 2, Model 1). Further, after adjusting for other confounding factors such as systolic blood pressure, smoking, and drug history (i.e., usage of statins or ARB or ACE inhibitors), arterial stiffness was significantly associated with increased level of serum omentin-1 (*P *for trends = 0.023; Figure 2, Model 2). Based on the results of multiple linear regression analyses, age (*P *< 0.001), systolic blood pressure (*P *< 0.001), medication history including statin (*P *= 0.007) and ARB or ACE inhibitor (*P *= 0.013) and serum omentin-1 level (*P *= 0.023) were independent determining factors for mean baPWV (*R*^2 ^= 0.637)

### Odds ratio of serum omentin-1 level predicting carotid plaque in type 2 diabetes

According to univariate and multivariate logistic regression analyses, the ORs of carotid plaque according to serum omentin-1 level are displayed in Table 2. Interestingly, the OR for carotid plaque was 0.692 (95% CI, 0.510-0.938) according to the increased level of serum omentin-1 based on an unadjusted model. The adjusted ORs for carotid plaques in the first, second, third and fourth models were 0.672 (95% CI, 0.478~0.944), 0.650 (95% CI, 0.454~0.930), 0.649 (95% CI, 0.438~0.962), 0.621 (95% CI, 0.420~0.919), respectively. Most importantly, inclusion of confounding factors which can affect carotid atherosclerosis did not alter the results of our multiple regression analyses.

**Table 2:** Univariate and multivariate logistic regression analyses for carotid plaques in type 2 diabetes

Variable	B	S.E	Odds ratio	P-value
			(95% C.I)	
Type 2 diabetes (N = 60)				
Univariate	-0.369	0.156	0.692 (0.510 ~ 0.938)	0.018
Multivariate				
Model 1*	-0.398	0.173	0.672 (0.478 ~ 0.944)	0.022
Model 2^†^	-0.431	0.183	0.650 (0.454 ~ 0.930)	0.018
Model 3^‡^	-0.432	0.201	0.649 (0.438 ~ 0.962)	0.031
Model 4^§^	-0.476	0.200	0.621 (0.420 ~ 0.919)	0.017

B, regression coefficient; S.E, standard error; C.I, confidence interval

* Adjusted for age and gender

^† ^Adjusted for age, gender and body mass index

^‡ ^Adjusted for age, gender, body mass index, systolic blood pressure, fasting blood glucose and low density lipoprotein cholesterol

^§^Adjusted for age, gender, body mass index, systolic blood pressure, fasting blood glucose, low density lipoprotein cholesterol, smoking and medication history including anti-platelet agents and statins.

## Discussion

We demonstrated that serum omentin-1 levels were significantly decreased in type 2 diabetes patients compared to normal glucose controls, and were further reduced in type 2 diabetes patients with carotid plaque compared to those without carotid plaque. Furthermore, the circulating omentin-1 value was an independent determining factor for arterial stiffness and the existence of carotid plaque in subjects with type 2 diabetes, even after adjusting for confounding cardiovascular risk factors and detailed medication history.

### Adipokines and atherosclerosis

The adipose tissues of individuals with type 2 diabetes secrete pro-inflammatory adipokines that contribute to the initiation of insulin resistance [24]. Some adipokines, such as resistin, leptin, and adiponectin directly mediate vascular health by influencing the function of endothelial cells, arterial smooth muscle cells, and macrophages in the vessel wall [3]. Recently, we reported that serum resistin and adipocyte fatty acid-binding protein are independently associated with atherosclerotic inflammation, as determined by [^18^F]-fluorodeoxyglucose positron emission tomography [25,26]. The production of adipokines by adipose tissue is an important mechanism for the adverse effects of adiposity on cardiovascular disease [27].

### Relationship of the novel adipokine omentin-1 with vascular health: Clinical studies

Omentin, which is highly and selectively expressed in visceral adipose tissue, has been suggested to be an anti-inflammatory molecule [28] and is thus assumed to have paracrine and autocrine roles in improving insulin sensitivity [7]. In 2011, several clinical studies examined the relationship between circulating omentin-1 with vascular health. Moreno-Navarrete et al. [11] demonstrated that the circulating omentin-1 concentration contributes independently to endothelial dysfunction even after controlling for adiposity, age, and inflammation in subjects with impaired glucose tolerance. In that study, vascular reactivity was measured by high-resolution ultrasound of the brachial artery. Since then, two other reports on the negative correlation of serum omentin-1 with carotid IMT have suggested an cardioprotective and anti-atherosclerotic role of omentin-1 [12,14]. Liu et al. [12] demonstrated that serum omentin-1 level was independently correlated with carotid IMT in metabolic syndrome patients and Shibata et al. [14] showed similar results in apparently healthy men. However, no studies have investigated the impact of serum omentin-1 level on atherosclerosis in type 2 diabetes. Recently, El-Mesallamy et al. [29] examined the circulating level of omentin-1 in an Egyptian population with type 2 diabetes, with and without ischemic heart disease. Although they did not detect clear differences in serum omentin-1 levels between type 2 diabetes patient with and without ischemic heart disease, multiple regression analysis showed that IL-6 level was an independent risk factor influencing serum omentin-1 level. This suggests that omentin-1 is regulated by inflammation. Inflammation is the most important factor linking type 2 diabetes to the progression of cardiovascular complication. Recently, Zhong et al. [13] demonstrated that serum omentin-1 levels were lower in patients with acute coronary syndrome or stable angina pectoris compared to controls. We showed that even after controlling for other risk factors and drug history that could affect vascular function, serum omentin-1 level was independently associated with arterial stiffness and carotid plaque. Previous studies and our results suggest that circulating omentin-1 might mediate not only the insulin signaling pathway but also directly affect the anti-atherosclerotic process.

### Underlying mechanisms of omentin-1 and atherosclerosis: Experimental studies

Recently, several *in vitro *studies have been published that could explain the underlying mechanism of the connection between circulating omentin-1 and the atherosclerotic process. Yamawaki et al. [30] found that, in the isolated rat aorta, omentin directly induces an endothelium-dependent relaxation that is caused by nitric oxide produced by the endothelium. The same group [31] also showed that omentin inhibits TNF-α induced COX-2 expression by inhibiting the JNK signaling pathway in human umbilical vein endothelial cells, suggesting that omentin might prevent atherosclerosis by modulating the vascular endothelial inflammatory state. Furthermore, Xie et al. [32] reported that adenovirus-mediated overexpression of omentin-1 attenuated arterial calcification in OPG^-/- ^mice, suggesting that increasing concentrations of omentin-1 may be beneficial for protecting arteries.

### Limitations

The principal limitation of our study was its cross-sectional design. We cannot identify whether increased circulating omentin-1 had a direct impact on arterial stiffness and carotid plaque or was a consequence of increased arterial stiffness and arterial wall thickness. Therefore, we are planning a longitudinal study of the relationship of circulating omentin-1 levels with changes in baPWV and carotid plaque in people with newly diagnosed type 2 diabetes. Second, we enrolled only a limited sample, so the relationship between circulating omentin-1 and atherosclerosis in type 2 diabetes should be further examined in a prospective design with a larger number of subjects. Lastly, although recent reports have demonstrated a close relationship between serum omentin and adiponectin levels, and that the regulation of omentin might be dependent on adiponectin [33], we did not measure adiponectin level. Further studies examining the influence of the relationship between omentin and adiponectin levels on cardiovascular disease would be interesting.

## Conclusions

In conclusion, this study showed that the circulating omentin-1 level is significantly decreased in type 2 diabetes patients compared with control subjects and further decreased in subjects with carotid plaque. We demonstrated that serum omentin-1 level was independently associated with arterial stiffness and the presence of carotid plaque, even after adjusting for other cardiovascular risk factors and medication history. Additional experimental studies and large-scale prospective clinical studies are warranted to clarify the role of omentin-1 on the atherosclerotic process in people with type 2 diabetes.

## List of Abbreviations

ARB: angiotensin receptor blocker; ACE: angiotensin-converting enzyme inhibitor; baPWV: brachial ankle pulse wave velocity; BMI: body mass index; DBP: diastolic blood pressure; IMT: intima-media thickness; HDL: high-density lipoproteins; HOMA-IR: homeostasis model assessment of insulin resistance; hsCRP: high sensitivity C-reactive protein; LDL: low-density lipoprotein; SBP: systolic blood pressure.

## Competing interests

The authors declare that they have no competing interests.

## Authors' contributions

HJY and SHB participated in the design of the study and performed the statistical analysis. HJY and SHB drafted the manuscript. HJY and SJY analysed the data. HCH, HYC, JAS, SGK, NHK, KMC and DSC conceived of the study, and participated in its design and coordination and helped to draft the manuscript. All authors read and approved the final manuscript.
